# Split fingernails, underdeveloped thumbnails, and triangular lunulae

**DOI:** 10.1016/j.jdcr.2025.02.009

**Published:** 2025-03-07

**Authors:** Erin R. Pomerantz, Nicholas D. Brownstone, Sylvia Hsu

**Affiliations:** Department of Dermatology, Temple University Lewis Katz School of Medicine, Philadelphia, Pennsylvania

**Keywords:** dystrophic nails, hereditary onycho-osteodysplasia, hypoplastic patellae, iliac bone exostoses, nail-patella syndrome, radial head dysplasia, triangular lunulae

## Case presentation

**Fig 1:**
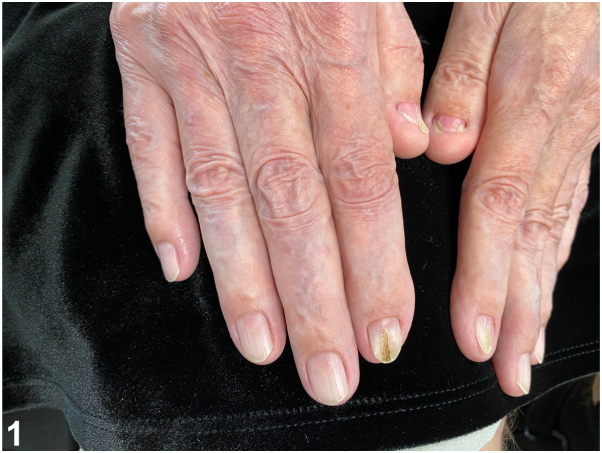

A 67-year-old man presented to the dermatology clinic for evaluation of split nails. His medical history was notable for bilateral elbow surgeries. On physical examination, it was noted that the patient had split nails of his index fingernails and underdevelopment of his thumbnails (Fig 1). All his fingernails, except for the thumbnails, had triangular lunulae.

**Question 1: What is the most likely diagnosis?**

A. Darier disease
B. Lichen planus (LP)
C. Nail-patella syndrome (NPS)
D. Pachyonychia congenita
E. Psoriasis

**Answers:**

A. Darier disease – Incorrect. Darier disease primarily affects keratinization, leading to hyperkeratotic and warty plaques in a seborrheic distribution. It also manifests with nail changes, including longitudinal red and white streaks, as well as distinct V-shaped notching of the distal nail plate, which may mimic the splitting and cracking seen in the nails of patients with NPS.
B. LP – Incorrect. LP is characterized by violaceous, pruritic papules, and plaques with Wickham striae. LP of the nail classically causes trachyonychia and dorsal pterygium.
C. NPS – Correct. NPS, also known as hereditary onycho-osteodysplasia or Fong disease, typically presents with abnormalities in the nails and the bones of knees, elbows, and pelvis. Nails may be absent, underdeveloped, split, or ridged with pathognomonic triangular lunulae. Bony abnormalities include absent or hypoplastic patellae, radial head dysplasia, and iliac bone exostoses.^1^
D. Pachyonychia congenita – Incorrect. Pachyonychia congenita presents with oral leukokeratosis, palmoplantar keratoderma, and thickened nails with subungual hyperkeratosis.^2^ In contrast, NPS is marked by dystrophic nails, triangular lunulae, and skeletal deformities.
E. Psoriasis – Incorrect. Psoriasis is a skin condition that characteristically presents with well-circumscribed, erythematous, and scaly plaques. Psoriasis can affect nails with varying presentations, including irregular pitting, oil drop spots, and onycholysis. Psoriasis affecting the nail does not present with triangular lunulae.

**Question 2: What systemic complication is seen in this condition?**

A. Cardiomyopathy
B. Chronic obstructive pulmonary disease (COPD)
C. Endocrine dysfunction
D. Nephropathy
E. Osteoporosis

**Answers:**

A. Cardiomyopathy – Incorrect. Cardiomyopathy can occur in various systemic diseases, including metabolic, inflammatory, and infiltrative conditions. Cardiomyopathy is not a typical feature of NPS.
B. COPD – Incorrect. COPD is most associated with a history of smoking and environmental factors that lead to respiratory dysfunction. NPS does not involve the respiratory system, nor is it known to be a cause of chronic pulmonary conditions, like COPD.
C. Endocrine dysfunction – Incorrect. NPS does not typically present with endocrine abnormalities. NPS is more commonly associated with ocular hypertension and glaucoma due to its effects on the eyes, as well as renal complications, like nephropathy.
D. Nephropathy – Correct. Renal involvement is seen in up to 40% of individuals with NPS. Renal involvement can range from nephropathy to end-stage renal disease.^3^
E. Osteoporosis – Incorrect. While NPS is associated with skeletal abnormalities, it does not typically cause osteoporosis. NPS is characterized more by structural abnormalities, like absent patellae, radial head dysplasia, and iliac bone exostoses. These features are more typical of NPS than bone density loss seen in osteoporosis.

**Question 3: How do you confirm the diagnosis of this condition?**

A. Biopsy of the affected nails
B. Genetic testing
C. Ophthalmologic evaluation
D. Renal ultrasound
E. X-ray of the knees

**Answers:**

A. Biopsy of the affected nails – Incorrect. A nail biopsy does not reveal any structural or immunohistochemical abnormalities in the epidermal basement membrane.^4^ Triangular lunulae and other nail abnormalities seen in NPS are primarily clinically diagnosed features. Diagnosis is based on these characteristic findings, along with genetic testing for mutations in LMX1B.
B. Genetic testing – Correct. NPS is an autosomal dominant condition and genetic testing for mutations in the LMX1B gene is the most definitive confirmatory test. When there is a positive family history and clinical findings, genetic testing most accurately confirms the diagnosis.
C. Ophthalmologic evaluation – Incorrect. While individuals with NPS are at an increased risk of open-angle glaucoma and ocular hypertension, ophthalmologic evaluation is not used to diagnose NPS. Ophthalmologic evaluation can help to monitor the development of ocular complications.
D. Renal ultrasound – Incorrect. While renal involvement is a feature seen in NPS, renal ultrasound is used to assess kidney structure and function, not to confirm the diagnosis. The ultrasound may reveal abnormalities, but it is not specific to NPS and is not the diagnostic test for this condition.
E. X-ray of the knees – Incorrect. While X-rays of the knee can demonstrate patella abnormalities (hypoplasia or aplasia of the patellae) and help support the clinical diagnosis of NPS, it is not the gold standard in definitively diagnosing NPS.

## Conflicts of interest

None disclosed.
